# Supplementary material for: Perceptions and Factors Influencing Eating Behaviours and Physical Function in Community-Dwelling Ethnically Diverse Older Adults: A Longitudinal Qualitative Study
Source: Nutrients. 2019 May 29;11(6):1224. doi: 10.3390/nu11061224 (PMC6627566; doi:10.3390/nu11061224)
Supplement: Supplementary file 1 [file nutrients-11-01224-s001.pdf]

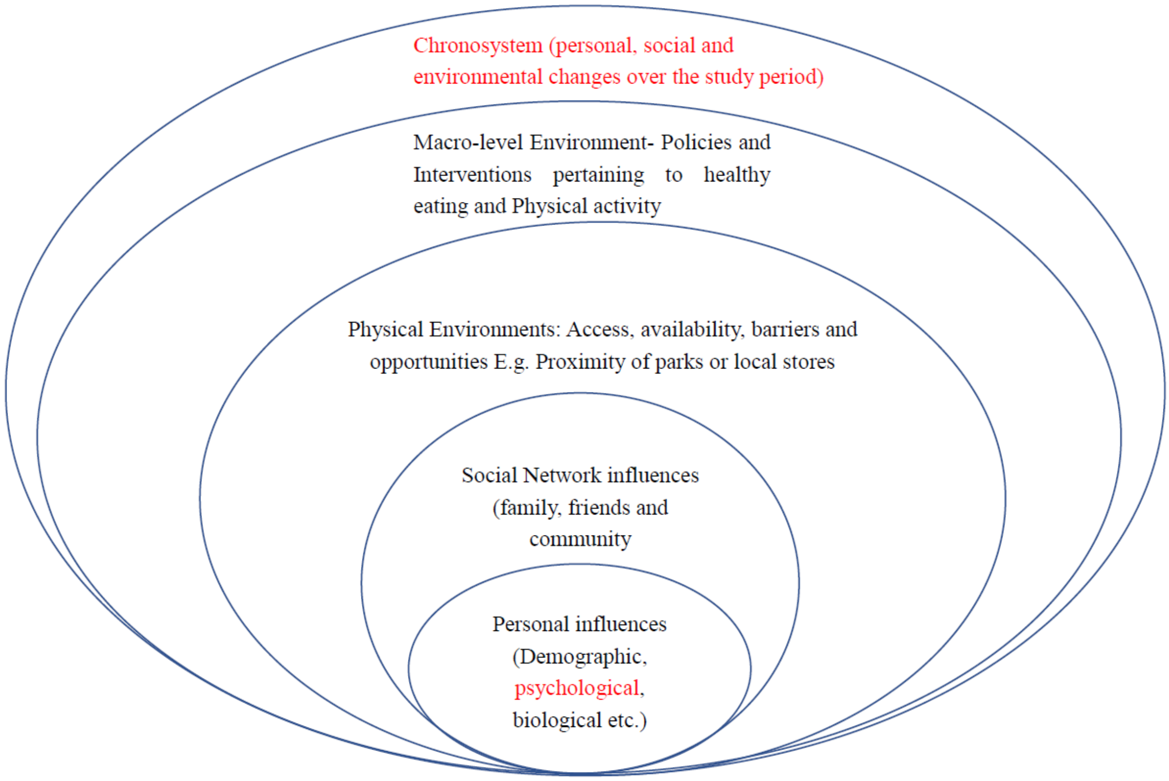

Figure S1. Adapted Ecological model depicting the influences of eating behaviours in community-dwelling ethnically diverse older adults.

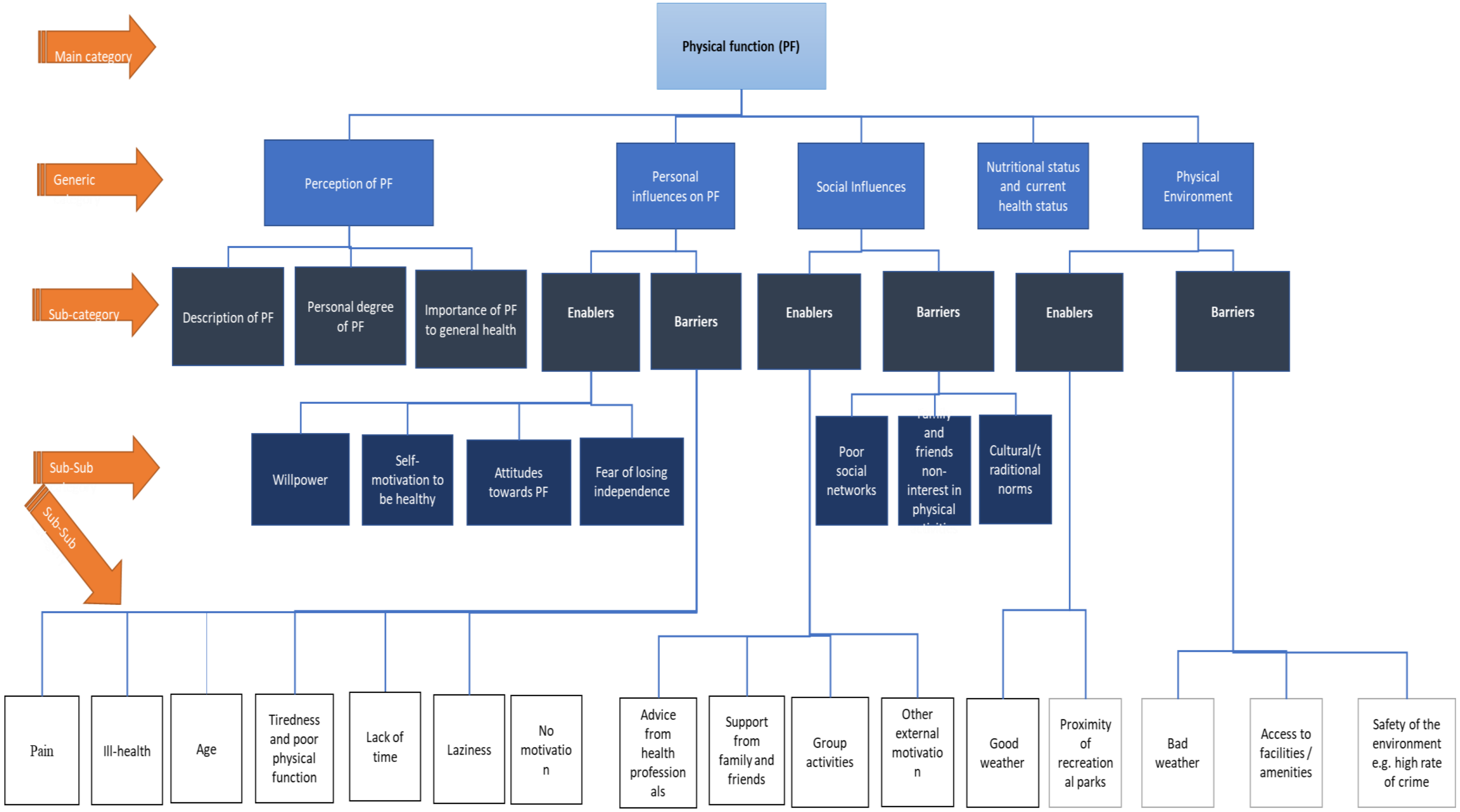

Figure S2: Complete coding matrix: Factors influencing physical function
